# A realist evaluation exploring simulated patient role-play in pharmacist undergraduate communication training

**DOI:** 10.1186/s12909-021-02776-8

**Published:** 2021-06-07

**Authors:** Aisling Kerr, Judith Strawbridge, Caroline Kelleher, James Barlow, Clare Sullivan, Teresa Pawlikowska

**Affiliations:** 1 School of Pharmacy and Biomolecular Sciences, RCSI School of Medicine and Health Sciences, RCSI University of Medicine and Health Sciences, 1st floor Ardilaun House Block B, 111 St, Stephen’s Green, Dublin 2, Ireland; 2 Division of Population Health Sciences, RCSI University of Medicine and Health Sciences, 123 St Stephen’s Green, Dublin 2, Ireland; 3 Department of Chemistry, RCSI University of Medicine and Health Sciences, 123 St. Stephen’s Green, Dublin 2, Ireland; 4 Department of Simulation, RCSI University of Medicine and Health Sciences, 123 St Stephen’s Green, Dublin 2, Ireland; 5 Health Professions Education Centre (HPEC), RCSI University of Medicine and Health Sciences, 123 St Stephen’s Green, Dublin 2, Ireland

**Keywords:** Communication, Interpersonal Communication, Patient-pharmacist communication, Pharmacy Education, Pharmacist, Pharmacy Student, Simulation, Simulated Patient, Realist Methods, Realist Evaluation

## Abstract

**Background:**

Effective communication between pharmacists and patients is essential and improves health outcomes. Simulated patients (SPs) are trained to reproduce real-life situations and can help pharmacy students to develop and adapt their communication skills in a safe, learner-centred environment. The aim of this research was to explore how SP and pharmacy student role-play supports communication training.

**Methods:**

A mixed methods realist evaluation approach was adopted to test an initial theory relating to SP role-play for pharmacy students. The intervention tested involved complex communication cases in a men’s and women’s health module in year three of a new MPharm programme. This SP session was the first such session, of the programme which exclusively focused on complex communication skills for the students. Data collected comprised video-recordings of both training and mock OSCE sessions, and from student focus groups. Communication videos were scored using the Explanation and Planning Scale (EPSCALE) tool. Scores from SP and mock OSCE sessions were compared using the Wilcoxon-signed rank test. Focus groups were conducted with students about their experience of the training and analysed thematically, through a realist lens. Data was analysed for Context-Mechanism-Outcome configurations to produce modified programme theories.

**Results:**

Forty-six students (*n* = 46/59, 78 %) consented to their video-recorded interactions to be used. Students identified contextual factors relating to the timing within the course and the setting of the intervention, the debrief and student individual contexts. Mechanisms included authenticity, feedback, reflection, self-awareness and confidence. Negative responses included embarrassment and nervousness. They distinguished outcomes including increased awareness of communication style, more structured communication and increased comfort. However quantitative data showed a decrease (*p* < 0.001) in communication scores in the mock OSCE compared with scores from training sessions. Modified programme theories relating to SP training for pharmacy students were generated.

**Conclusions:**

SP role-play is a valuable communication skills training approach. Emphasis should be placed on multiple stakeholder feedback and promotion of reflection. Time limits need to be considered in this context and adjusted to meet student needs, especially for students with lower levels of communication comfort and those communicating in languages different to their first language.

**Supplementary Information:**

The online version contains supplementary material available at 10.1186/s12909-021-02776-8.

## Background

Effective communication with patients is essential and improves health outcomes [1]. Pharmacy competency frameworks and the World Health Organisation emphasise communication as a core competency for pharmacists [2–4]. The role of pharmacists has expanded over recent years to a more clinical, patient-centred one, making communication an essential aspect of daily practice [5], and pharmacists need to be able to adapt their communication style to a wide variety of patient needs [6]. Pharmacy education standards highlight the importance of communication training and communication in practice [5, 7–9]. Communication skills develop through complex interactions during undergraduate pharmacy education, including via experiential learning and communication skills training [5, 10].

Simulation is a safe, learner-centred educational approach that exposes trainees to various levels of complexity, similar to real experiences, with an adjustable level of challenge [11]. Simulated patient communication training can help to equip future pharmacists with the necessary skills to communicate with and adapt their communication to individual patients. SPs are trained to replicate real life situations for the purpose of training or assessment [7, 12] and are trained to provide constructive feedback, which is a key contribution to student learning [12, 13]. The benefits of SPs are allowing students the reality of experience without placing actual patients at risk and allowing students to learn from their mistakes [11, 12]. The majority of studies relating to health professions communication training using SP are focussed in medicine [11, 14], although studies are emerging in pharmacy [7, 15, 16].

A realist approach is particularly useful for this research as there is little understanding of how and why communication training interventions for pharmacists lead to outcomes. We feel it is important to look beyond the intervention itself using realist methodology because communication skills training is a dynamic intervention and multiple context-dependent components are involved. Realist research works on the philosophy that the same intervention, when applied in different contexts, which may be external or internal, can trigger different mechanisms and may lead to different outcomes [17]. This realist evaluation aimed to test an initial realist programme theory, which was developed from a realist synthesis completed by the research team. The realist synthesis explored how interventions to develop interpersonal patient-pharmacist communication skills produce their effects [18]. The realist synthesis produced realist programme theories relating to how peer, faculty and simulated role-play, video-recording, workshops and self-assessment interventions work to develop pharmacist communication skills [18]. This realist evaluation tests the realist programme theory relating to video-recorded simulated patient role-play [18]. This initial realist programme theory was ‘*video-recorded role-play with SPs works for intermediate pharmacy students through practice and feedback to improve communication through reflection and self-awareness’*.

### Objectives

**Fig. 1:**
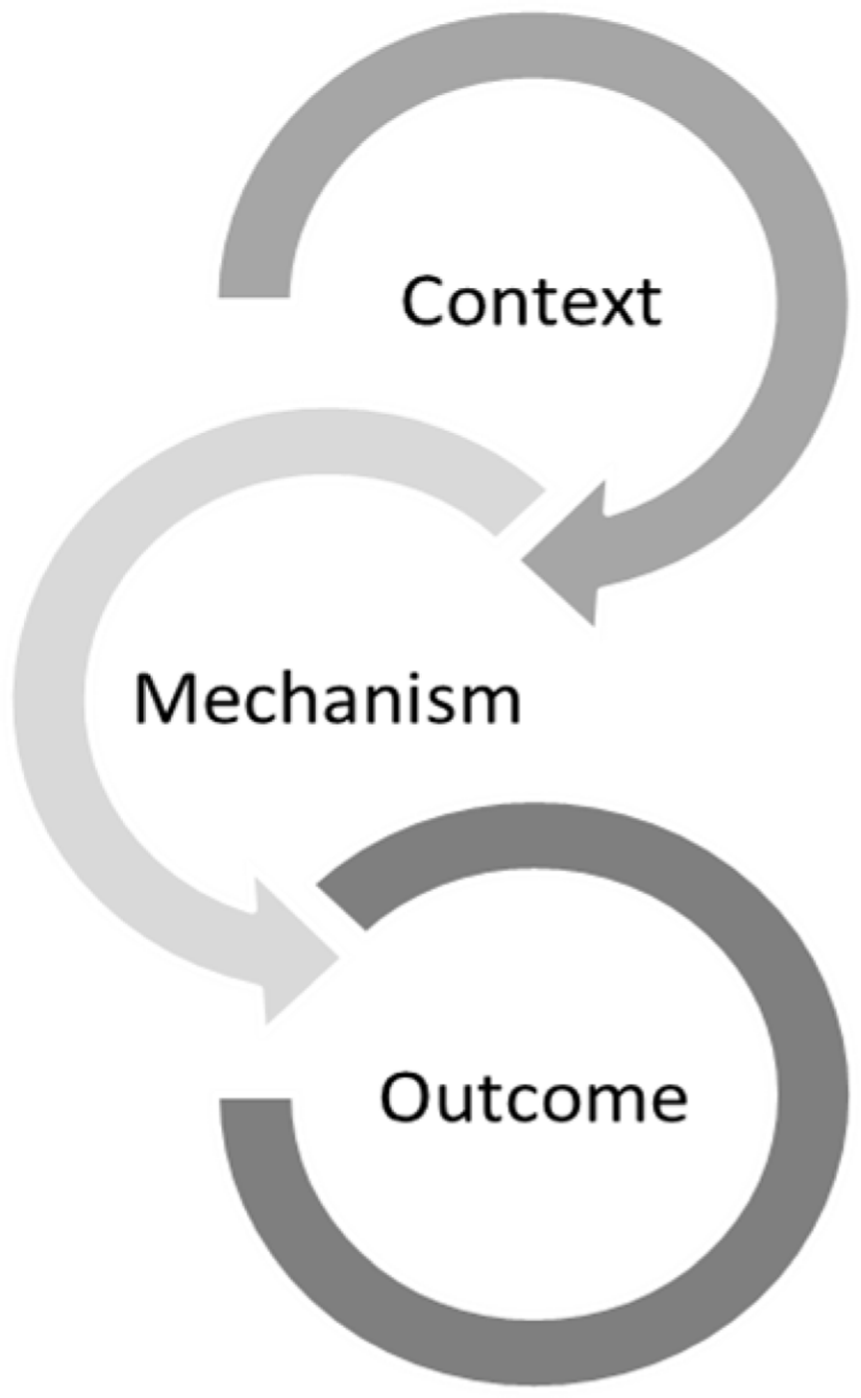
Context-Mechanism-Outcome Configurations are a Key Component of Realist Evaluation

This realist evaluation asked: How does simulation work to train pharmacy students in interpersonal patient-pharmacist communication? The study aimed to explore the contexts and mechanisms by which SP training to develop interpersonal patient-pharmacist communication skills support their intended outcomes (See Fig. 1).

## Methods

### Study design

This was a mixed methods realist evaluation following course reform. Realist methods are a relatively new and evolving approach. Realist research explores the links between contextual factors and the processes or mechanisms these trigger, to explain why and how different outcomes have been achieved [19, 20]. The methods for realist evaluation are defined [19, 21]. Realist research explores the links between contextual factors and the processes or mechanisms these trigger, to explain why and how different outcomes have been achieved [17, 19, 22]. Realist research asks “*what works, for whom, in what circumstances, in what respects, to what extent and why*?” [20]. Realist evaluations consist of four stages: development of initial realist programme theories, gathering evidence, configuring evidence, and refinement of realist programme theories [19]. Realist evaluations are iterative by nature and require initial realist programme theories to be revisited and refined after each data collection point [19].

Context-Mechanism-Outcome configurations are a key component of data synthesis in realist research. These are links of what mechanisms are triggered in certain contexts and what outcomes arise from these. Contexts may be related to the setting or individual participants. Mechanisms may be described as resource or response. Resource mechanisms come from the intervention itself and response mechanisms relate to participants’ reaction to the resources [17]. Quantitative data consisted of communication scores from video-recorded interactions. Qualitative data came from student focus groups. The initial realist theory tested, ‘video-recorded role-play with SPs works for intermediate pharmacy students through practice and feedback to improve communication through reflection and self-awareness’ was developed by a completed realist synthesis [18].

### Setting and Intervention

This study examined a SP session in a men’s and women’s health module. This session took place in the first semester of year 3 of a new 5-year MPharm programme. The new MPharm curriculum was introduced in RCSI in 2015, as part of a major curriculum reform and change in pharmacy education in Ireland [8, 23]. Experiential learning and SP contact approaches were integrated as part of the new programme. Communication skills training in years one and two focusses on an introduction to communication, including basic skills, structuring communication using the Cambridge-Calgary model [24], and basic interactions. Training is primarily delivered through patient-centred care laboratories, peer-role play and mock counselling with faculty in patient-centred care labs and through public and patient involvement throughout all modules. Year two students complete a longitudinal community pharmacy placement. Students in year two have one formative and one summative Objective Structured Clinical Examination (OSCE), which includes a formal assessment of communication skills. The aim of communications skills training in year three is for students to develop more complex communication skills. Building on high-fidelity simulation with manikins in year 2, increased use of SPs was deemed appropriate for teaching these more complex communication skills. Therefore, in order to maintain patient interactions, it was imperative to increase SP contact and this was done through pragmatic integration of communication skills training into each module. Prior to the men’s and women’s health module, students completed Mental Health First Aid Training and real patient interactions in a hospital setting. The SP training in the men’s and women’s health module was the student’s first exposure to SP role-play exclusively focused on complex communication skills, which was part of the pragmatic construction of SP contact. Other communications skills training in year three following the formative mock OSCE include interprofessional learning and SP training in a cancer module. Therefore, due to previous communication skills training and experiential learning, these year three students were classified as ‘intermediate’.

The training intervention was designed for students to practice and receive feedback on a variety of complex communication scenarios relevant to pharmacy practice. Cases included a pregnant woman seeking smoking cessation advice or who had recently consumed alcohol, the use of tinzaparin in pregnancy, the requesting of emergency hormonal contraception, counselling on commencement of sildenafil therapy for erectile dysfunction and a man seeking advice on potential teratogenicity of a new medication. The year three class was split into randomly assigned small groups of 12 students for the training session, with students divided into six pairs. Sessions took place in a purpose-built simulation centre. During a session, students rotated through six scenarios in pairs, with students taking it in turns to act as pharmacist and observer i.e. for each scenario one student acted as the pharmacist and one student observed the interaction. The students alternated these roles for every second scenario, until all six scenarios were completed. There was a time limit of five minutes for each interaction. The scenarios were written and designed with the intention that they would be completed within the assigned time limit. There was a two-minute turnaround time between scenarios, during which time the SP exited the room, prior to the entry of a new SP for the next scenario. Directly following completion of all six simulated role-play scenarios, students regrouped with their small group, the faculty members facilitating the session and SPs in a small tutorial room, with movable chairs for a debrief session. During the debrief session, all students in the small group watched one video of each student’s interaction. The communication in the performance was discussed in the small group and feedback was provided by the faculty members, peers in the group and SPs.

**Fig. 2:**
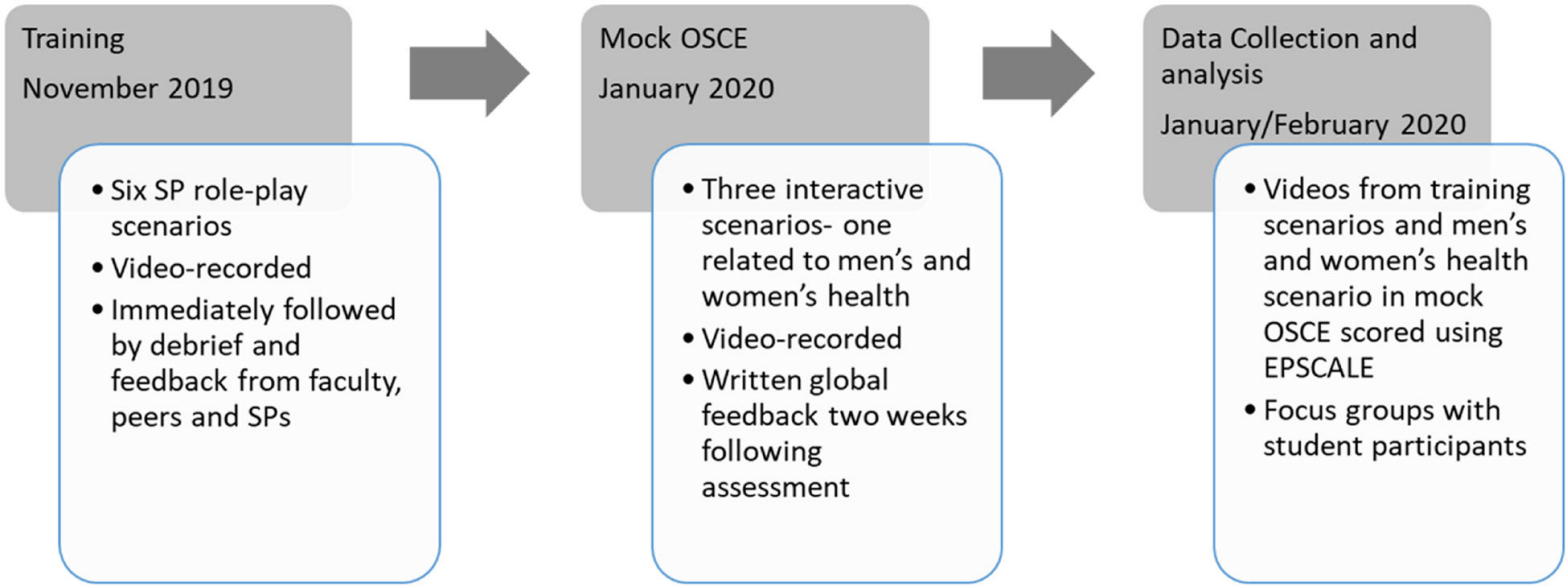
Study flow of intervention, formative assessment and data collection

Training sessions were held in November 2019, on five occasions over a 4-week period, with students attending on one day only, to allow for all students to attend training. Attendance at a training session was compulsory for all students within the men’s and women’s health module. The scores from the videos recorded during the training sessions were considered as the baseline results (Fig. 2).

The formative, mock OSCE examination was held in January 2020, in the same venue as the training sessions. It consisted of three interactive stations, with one relating to the men’s and women’s health module and covering the treatment of genital warts. The other stations related to a liver and kidney module and an endocrine system module. As these scenarios were considered non-equivalent, only the scenario on genital wart treatment was included in this study. The time limit for the scenarios was also five minutes and all students completed each station, with no peers present. A SP and a faculty assessor were present in the room during the interaction. Global written feedback was provided two weeks following the formative assessment. See Fig. 2 for a summary of the flow of the intervention, formative assessment and data collection in this study.

### Data Collection and analysis

Video recordings of the interactions from the SP session and the equivalent mock OSCE case were collected in CAE LearningSpace®, a clinical simulation management platform. As students were taught using the Cambridge-Calgary Guides [24], scoring was aligned to this structure. Therefore, videos were scored using the the Explanation and Planning Scale (EPSCALE) instrument [25], a scale for teaching and assessment of explanation and planning skills used by clinicians during the medical interview, (Table 1) [25]. All videos were scored by AK and 10 % of the videos were scored independently in duplicate by TP and discussed for standardisation.

**Table 1:** EPSCALE tool score descriptors

EPSCALE	0	1	2	3
**Building the relationship**				
**Respects patient**	Shows no interest or concern OR is overtly offensive	Little interest and concern for patient’s well being	Some interest and concern for patient	Clear interest and concern for patient as a person
**Empathy**	Ignores patient’s feelings and predicament	Minimal (only non-verbal) response to patient’s feelings and predicament	Some verbal response to patient’s feelings and predicament	Sensitive verbal and non-verbal response to patient’s feelings and predicament
**Uses appropriate non-verbal behaviour**	No eye contact OR inappropriate non-verbal behaviour	Little eye contact OR some inappropriate non-verbal behaviour	Good eye contact, generally appropriate non-verbal behaviour	Good eye contact, substantial and appropriate non-verbal behaviour
**Providing the correct amount/type of information for the individual patient**				
**Chunks and checks, using patient’s response to guide next steps**	Gives long, uninterrupted speech	Occasional pauses but does not elicit patient’s response	Pauses, with some effort to gauge patient’s response before proceeding	Repeatedly chunks and checks, using patient’s response to guide next steps
**Assesses the patient’s starting point**	No attempt to gauge patient’s starting point	Attempts to find out starting point but still gives info as prepared	Discovers starting point, some adjustment to info-giving	Discovers starting point and patient’s preference for amount of information, carefully tailor’s explanation
**Discovers what other information would help patient**	No effort to discover what extra information would help	Little effort to discover or respond to patient’s info needs	Makes some effort to discover and address patient’s info needs	Carefully and repeatedly seeks and addresses patient’s needs
**Aiding accurate recall and understanding**				
**Organises explanation**	No organisation of explanation	Minimal organisation of explanation	Organises explanation, but no overt signposting/summarising	Organises explanation, with overt signposting/summarising
**Checks patient’s understanding**	Does not check patient understanding	Minimal checking that patient has understood	Carefully checks that patient has understood	Asks patient to restate information given
**Uses clear language**	Frequent use of unexplained jargon and confusing language	Some unexplained jargon and confusing language	Majority of language used clear (1–2 unexplained jargon words only)	Clear language used throughout
**Achieving a shared understanding: incorporating the patient’s perspective**				
**Relates explanations to patient’s illness framework**	No reference at all to patient’s ideas, concerns, expectations	Little attempt to relate explanation to patient’s ideas, etc.	Makes reasonable attempt to relate explanation to patient’s ideas, etc.	Sensitively relates explanation to ideas, etc.
**Encourages patient to contribute reactions, feelings and own ideas**	No opportunities for patient to contribute	Limited opportunities for patient to contribute but no response	Several opportunities for patient to contribute with some response	Actively encourages patient to contribute and responds well
**Picks up & responds to patient’s non-verbal & covert verbal cues**	No response to patient’s non-verbal and covert verbal cues	Minimal response to patient’s non-verbal and covert verbal cues	Some response to patient’s non-verbal and covert verbal cues	Sensitively responds to patient’s non-verbal and covert verbal cues
**Planning: shared decision making**				
**Explores management options with patient**	No exploration of available options, only directives given	Offers options in cursory fashion	Carefully explores options with patient	Fully explores options and dilemmas, signposting position of equipoise or own preferences
**Involves patient in decision making**	No involvement or resists involvement of patient in decision making, directives given	Makes suggestions rather than directives but limits patient involvement in decision making	Actively encourages patient involvement in decision making	Establishes level of involvement patient wishes in decision making: if appropriate, fully encourages patient to make choices & decisions
**Appropriately negotiates mutually acceptable action plan**	Presents plan without checking with patient	Presents plan with cursory check for patient’s approval	Reasonable and appropriate negotiation of plan with patient	Full and appropriate negotiation of plan with patient; final agreement checked

Statistical analyses were conducted using Stata SE/16.0. Descriptive statistics were used to summarise student demographics and the number of scenarios completed by students. Scores from the SP session and mock OSCE were compared using the Wilcoxon-signed rank test. Mean scores for each case were used to compare performance across cases. No adjustment for multiple comparisons was conducted as this was an exploratory study and sample sizes were small.

In order to gain further insight into how and why SP training may work for communication skills training, qualitative focus groups were conducted with students, with 3–5 students per focus group. The focus group topic guide was designed in a realist interview manner, to facilitate open discussion about the respondents’ experiences of the programme [26]. Realist interviews are conducted with a focus on what works for whom, how and why, in order to gain detail on contextual factors and mechanisms that may contribute to the outcomes of an intervention. This approach was important to gather data to support the testing and refining of the realist initial programme theory [26]. The focus group topic guide is available in Supplemental Information One. There was a maximally variant sample frame, achieved through a gender, age and nationality balance of student participants. Focus groups were conducted until it was agreed by the research team that saturation of major themes for initial programme theory refinement was reached. Saturation of major themes for initial realist programme theory refinement included saturation of individual student contexts, contexts relating to setting, resource and response mechanisms and outcomes.

All focus groups were audio-recorded and transcribed verbatim. Thematic analysis was conducted [27], with a specific focus on realist programme theory testing, refinement and development. The initial programme theory relating to simulated patient-role play, from the previous completed realist synthesis [18], was tested and refined through the thematic analysis. The focus groups were imported into NVivo Version 12 to aid coding, by the first author, in collaboration with the research team. Coding was done iteratively in multiple stages to identify and further explore prominent themes as they emerged from the data. Codes were classified as context, mechanism, outcome or barriers. This coding was agreed upon by the research team to ensure they reflected the data.

 This study was conducted in accordance with ethical approval granted by the RCSI Human Research Ethics Committee. All students who consented for their videos to be used in this study gave written informed consent prior to the training session. All student participants in the focus groups provided written informed consent prior to the beginning of the focus group.

## Results

### Quantitative

46 students (*n* = 46/59, 78 %) consented for their video-recorded interactions to be used in this study. Their mean age was 22 years (at time of completion of mock OSCE assessment) (Standard deviation +/- 2.8 years). Most of the participants were female (*n* = 37, 80 %) and of EU nationality (*n* = 32, 70 %). Table 2 shows the number of cases completed by each student. The majority of students completed both the training session and the mock OSCE assessment (*n* = 43, 93 %) and a small number of students who completed the intervention did not attend the mock OSCE (*n* = 3, 7 %).

**Table 2:** Number of training stations completed

Number of cases completed	Number of students
5 training + mock OSCE	2
4 training + mock OSCE	1
3 training + mock OSCE	14
3 training only	2
2 training + mock OSCE	23
2 training only	1
1 training + mock OSCE	3

The alpha coefficient for each item on the EPSCALE was high, with low covariance: alpha coefficient for the overall scale was 0.95, covariance was 0.14. There was no case variability in reliability of the scale or inter-item covariance identified.

The mean score for each domain across all cases and all students in the training cases was 2.07 out of 3. The average score for the mock OSCE was 1.88. Table 3 illustrates the mean score for each individual station out of 3.

**Table 3:** Mean score for each station

Case	Mean Score (+/- SD)
Erectile dysfunction- sildenafil prescription	2.06 (0.49)
Alcohol/ Smoking in pregnancy	2.08 (0.45)
Teratogenicity	2.25 (0.46)
Emergency hormonal contraception (EHC)	2.04 (0.56)
Tinzaparin counselling in pregnancy	2.04 (0.44)
All training cases	2.07 (0.41)
Mock OSCE	1.88 (0.38)

There were significant decreases of the mean score (*p* < 0.001) in the mock OSCE compared with the overall mean scores from the training sessions.

### Qualitative

A total of four student focus groups were conducted, which identified a number of contextual factors, mechanisms and outcomes.

### Contextual Factors

Students felt that the timing of the intervention within the new curriculum was ideal, as the students knew each other and had good background knowledge, helping to create a safe environment. The SP sessions took place early in the module, increasing the focus on communication rather than knowledge.

The set-up of the consultation rooms helped to promote a safe environment for students. Students liked ‘*that it was a private room’*. The positioning of the cameras on the ceiling was good for students even though *‘we had clocked the three of them (cameras) as we went in’* some also mentioned they *‘kinda forgot the camera was there’.* Students liked the assigned pairs, rather than choosing their partner, as for them it created a professional environment.

Regarding the debrief following the SP session, students felt comfortable in the small groups and in the classroom. Students felt that that debrief *‘was done in a very lighthearted way’*, further promoting open conversation in a positive, comfortable environment. Students felt that the addition of SPs to their communication training made it ‘*more official’*, because they felt it was more like real-life not knowing the SPs as peers or teachers. However, knowing that they were not real patients also promoted a safe and controlled environment. Expectations of recording created somewhat of an uncomfortable context. For some students, the unknown nature of the case content in advance caused discomfort.

In relation to individual contexts, most students described feeling ‘*comfortable but not confident’* in communication prior to the session. Some students had worked in pharmacies before or in other part-time jobs and any previous work experience helped with baseline comfort in communication. Some students who are shy felt uncomfortable being videoed. For students who did not have English as a first language, they felt they did not have a natural flow with English communication. Illustrative quotes pertaining to contextual factors are included in Table 4.

**Table 4:** Illustrative quotes of contextual factors identified

**Timing of Intervention**	
‘I suppose in first year if you'd asked me to do something like that, I'd have been terrified. I'd have been so stressed it beforehand’.	
‘It was really early in our module. So, I kind of didn't expect to know anything, which then probably made the communication the most important’	
**Setting of Intervention**	
‘It was interesting though that we were assigned to those pairs, I thought, rather than people picking’	
‘The way the chairs were set up as well, I think it facilitated conversation…you weren't over the other side of the room and you weren't having to try and move closer.’	
**Debrief**	
‘everyone was positive about it, which was nice….any comments that were thrown out by the class were nice and positive…and then you kind of pick apart a bit more what could have been done different[ly]’	
‘it's a safety thing to have the SP because at least if you recommend something that is inappropriate, or possibly medically unsafe, then there's not going to be real life consequences I mean, and you can learn from the mistake’	
**Individual**	
‘You're more exposed to patients when you work in pharmacy…. Especially if you're OTC, like you're talking to patients all the time’	
‘I am shy, I don't want people to see my videos…so kind of like embarrassed’	
‘English is not my first language. So, kind of in my mind I have to arrange the sentence to say to the patient, but as years pass, I'm being more confident of communicating with patients’	

### Mechanisms

A number of resource mechanisms that came from the intervention were identified as potential ways through which SP training works. Students felt that the training challenged them more than interacting with peers or faculty, and was another opportunity within their course for practice. The key resource mechanisms involved are practice, authenticity, feedback, videos and fixed time. Illustrative quotes for resource mechanisms are listed in Table 5.

**Table 5:** Illustrative quotes from focus groups for identified resource mechanisms

**Practice**	
‘practicing more with the actors and just practicing more with communication skills in general’	
‘Thinking on your feet and just kind of, you know, winging it in….a structured way but good in that sense, and a good practice for a real life situation’	
**Authenticity**	
‘And it's more, it's kind of more, based on real life kind of scenario...like this is a typical day in day that you expect in a pharmacy’	
‘You weren't expecting, or you just had no idea what was going to be thrown at you, in terms of the scenarios.’	
‘I think having somebody that you don't know, so any sort of simulation, actors or patients does help to mirror that real-life situation’	
**Feedback**	
‘Any feedback they had, if even if it were, if even if it wasn't saying oh well done, you did this really well, it was delivered in a more constructive way’	
‘you really got to pick everything apart and you were getting different people's kind of opinions on how they saw a scenario go’	
‘The different perspectives…rather than how you felt subjectively it went. You were getting different angles on…perhaps you could have communicated this in a better way or from the patients in particular; I thought that was really good because they were able to say how they felt in general about how people were actually talking to them’	
**Time**	
‘It's good practice to learn how to focus your point and deliver key messages...ideally it'd be great if you went through everything with every patient but you'll only get through about 10 patients in the day’	
‘No, it's not practical, but at the same time it's good practice as much as you like not rush it or have it timed but like you don't want to be spending like an hour with every single patient.’	
**Videos**	
‘The videos at the end…helped you kind of look at…in retrospect how you performed in the videos like what areas you can improve on and why you can't improve’	

A number of response mechanisms, which are reactions triggered in an individual, were identified for how SP training works in pharmacy students. The main response mechanisms described as being involved are reflection, self-awareness and confidence. Negative responses described by students included embarrassment and nervousness, both of which subsequently triggered reflection. Illustrative quotes relating to response mechanisms are included in Table 6.

**Table 6:** Illustrative quotes for identified response mechanisms

**Reflection**	
‘Finding things in ourselves that we didn't even know were there before…It really helps you, to go back and reflect…it really changes your future interactions with other people’	
‘I suppose it was good to see your own sort of performance and then kind of comparing it to how other people did and then kind of learning from them and that’	
‘to kind of focus yourself in the video and see like if you look at it objectively and just look at, okay, what did I do right? What did I do wrong?’	
**Self-awareness**	
‘That helped, be a little bit more conscious of behaviors. Um, even if that's subconsciously at the back of your mind’	
‘I've noticed if I were particularly uncomfortable in the situation that I did fidget with my hands more…it was just kind of to note that thinking hang on, that's not going to put the patient at ease if I'm there fumbling with stuff’	
**Confidence**	
‘it's really beneficial in terms of just bringing up your confidence’	
**Embarrassment**	
‘I mean it was a bit embarrassing, but I think it showed habits that you might not have been aware’	
**Nervousness**	
‘The nerves of actually watching yourself back, but I think ultimately in the long run it was probably good because you picked up on things’	

### Outcomes

Through debrief and reflection, students became aware of the importance of keeping communication simple and of listening. Watching the videos made students aware of the need to plan what to say and how to say it. Another key outcome from the debrief was realizing the importance of building rapport with patients. Other benefits of the SP training described included more structured communication and awareness of patients’ emotions. They also felt more aware of their own tone of voice and body language. Illustrative quotes are in Table 7.

**Table 7:** Illustrative quotes for outcomes

**Listening**	
‘it shows you to listen a bit more when they're talking rather than constantly thinking what's next.’	
**Planning how to communicate**	
‘Actually, just wait a minute. Just even wait a second. Just say hang on to yourself and then think of a better way of framing things.	
**Aware of patient emotions**	
‘I can tell that this person is nervous. Wary by the way that they're acting or the tone of their voice’.	
**Self-awareness**	
‘be a little bit more attentive…in terms of altering tone of your voice or the way maybe how far you lean on a table or how much eye contact to make’.	
**Building rapport**	
‘We kind of learn a lot about building rapport with patients so they're comfortable telling your stuff but...We all skipped, we all just focused on the fact that we didn't know what the drug was.’	

### Barriers

Students perceived some barriers to their learning: the major one being the five-minute time limit per consultation. Students believed this inhibited their learning in multiple ways. In some cases, students felt time pressure meant that they got ‘*lost in trying to give them (patients) information that they were looking for’* and that the conversation was only beginning to flow *‘I was just trying to get the patient to open up and any then it was like time is up’*. Time limitations also created barriers to authenticity, as *‘different patients require different amounts of time.’*

The use of ‘props’, acted as a barrier for some students, such as a detailed patient information leaflet distracting students from actually communicating information to patients, as they got distracted by reading the leaflet. In some cases, such as the emergency hormonal contraception case, the lack of paperwork props such as an emergency hormonal contraception consultation form, which is completed in practice, and the time limit reduced authenticity for students ‘*I think the fact because you expected in real life to document a patient’s use of the emergency contraceptive pill the fact that that wasn’t included’.*

Students reported that the presence of a faculty assessor was a distraction and so felt to be a barrier to communication in the mock OSCE and moved the focus towards knowledge demonstration *‘you’re always looking…out the side of your eye to see if they’re, [the assessors are] ticking much or not ticking much’.* Students described this as discomforting and perturbing, particularly after the SP training was without having any faculty in the room: *‘then just to be suddenly back into that. It was sort of overwhelming. It’s not necessarily the most conducive to having a patient pharmacist conversation’.*

### Modified Programme Theories

The reported data was synthesised with a focus on realist initial programme theory refinement and it was determined that multiple modified realist programme theories, context dependent and relating to different outcomes, were needed. The modified realist programme theories do not extend to improved communication performance as quantitative findings do not provide evidence for this but serve to explain how SP role-play interventions produce their intended benefits.

The overarching modified realist programme theory is: ‘Video recorded role-play with SPs followed by debrief, works for intermediate pharmacy students through practice and authenticity, with feedback, reflection, self-awareness and confidence facilitating development of more structured communication, increased awareness of own communication style, communication comfort and confidence.’

Other emergent realist programme theories relating to more specific context-mechanism-outcome configurations include:

- For shy students, with low levels of communication comfort, video-recorded role-play works through practice, videos, and initial embarrassment and nervousness to lead to reflection to improve communication structure and awareness of communication style.
- For intermediate pharmacy students, who do not have English as a first language, video-recorded role-play with SPs works through practice, authenticity, feedback, nervousness, reflection and self-awareness to improve their communication structure, comfort and awareness of communication style. However, insufficient time may limit authenticity.
- At an early stage of a module, with pharmacy students having little case-specific background knowledge, role-play with SPs works through authenticity, practice, and feedback leading to reflection to improve rapport building and awareness of communication style.
- Debrief from faculty, peers and SPs following SP role-play works with small groups through feedback, reflection, self-awareness and confidence to improve communication structure.

A separate modified realist programme theory for the conditions of the mock OSCE to begin to explain the lower communication scores is as follows:

- The presence of a faculty assessor for formative assessment of SP role-play increases knowledge focus and creates discomfort, and limits authenticity but it provides opportunity for practice and reflection.

## Discussion

This study has tested and developed a modified realist programme theory explaining the contexts and mechanisms by which SP role-play produces outcomes in facilitating communication skills for intermediate pharmacy students. It was identified that communication performance declined in the formative OSCE compared to in the training sessions, potentially attributable to examination stressors including the presence of a faculty assessor during the formative OSCE interaction. This apparent decline may also be as this was a single communication skills training session and a single session may be insufficient to promote retention of communication skills improvement.

### Feedback

The value of feedback from multiple stakeholders is well described [28–32]. SP feedback on communication skills was particularly appreciated by pharmacy students to understand how they made the patients feel. This is in line with literature, which reports that SP feedback increases learner satisfaction and their relational focus [29, 30]. Peer feedback can be valuable, and the study identified the need to be familiar with classmates before this occurs [31, 33]. The role of faculty feedback is to provide more focused feedback, on clinical knowledge and relational aspects, in a structured manner [31, 34]. Others also report that feedback must be constructive and useful to the learner, and importantly, it confirms the need for support and reassurance when students receive feedback [32].

### Reflection

Reflection is a key response mechanism and was described by all focus group participants. The prominence of reflection as a mechanism is in line with the initial realist programme theory and findings from the previously conducted realist synthesis [18] and previous systematic reviews [5, 7, 35]. The links between reflection and self-awareness are well described in pharmacy education for the development of competencies and professional skills, including communication skills [36–38]. The outcomes and modified realist programme theories are in line with existing evidence that awareness of one’s own communication style and approach to communication can be improved through reflection [36–39].

### Scoring

In this study, communication scores averaged 69 % in the training sessions and 63 % in the mock OSCE. Levels of evidence for communication skills training in pharmacy have to-date largely focussed on perception data. Thus, studies that illustrate student communication scores during SP sessions are limited. A previous study, which explored the impact of SP training on pharmacy student communication, reported baseline scores of 43.6 %, a midpoint of 51.5 % and a final score of 53.7 % [40]. The scores of the students in this study could be seen as commencing from a higher baseline (bearing in mind training in earlier years of this course), with a resulting threshold effect and some temporal attrition affecting the drop in communication scores in the mock OSCE.

It was expected that students’ communication scores would be higher in the mock OSCE than the training sessions due to the debrief and time to practice and reflect. OSCE examinations, even if formative, are stressful and trying for students [41, 42], and may have adversely influenced student communication skills in the mock OSCE. The students treat the mock OSCE as an exam, they mentioned preparing for ‘the exam’, perhaps due to their high-achieving nature and competiveness. They also referred to the training session as an OSCE in the focus groups. This implies misunderstanding of the intent of the SP training and assessment for learning and hence the position of the mock OSCE, for formative feedback and exam practice. Furthermore, a previous study on the presence of faculty during communication simulation for students found that anxiety scores were significantly higher from pre-test to post-test when faculty were present in the simulation room [43]. This may in part, explain the apparent reduction in communication scores in the mock OSCE. However, as multiple variables were at play, direct comparisons ideally focussing on a single variable are needed to explore the impact of these.

### Time

Time acted a potential barrier and mechanism for this intervention. Literature suggests that time limits promote information-focus rather than patient-focus [44, 45] and it also creates an environment where students feel nervous [46]. Lack of time limits have been perceived by students, in other studies, to be more authentic [46, 47]. Studies reporting SP training for pharmacy student communication development do not always report duration of the consultation, but have ranged from thirty seconds to fifteen minutes [7]. There is, however, limited reporting of how long a typical patient-pharmacist consultation should last [7]. Community pharmacy consultations often require diagnosis, product recommendation and counselling on the product’s appropriate use, of various complexity [48]. A previous study in the US has advocated for at least three minutes per patient, with additional time added, depending on the complexity of the interaction. The previous study advocated for 1.8 min additional time relating to therapeutic appropriateness, 2.5 additional minutes to refer, with a range of additional time from 2.9 to 9 min [49]. This suggests that the 5-minute time limit of the described iteration of this intervention was likely insufficient for most of the interactions. In light of the findings of this study, the time limit of five minutes in the described iteration of the training sessions has been increased to seven minutes for the next iteration of this intervention, which was delivered online, due to the covid-19 pandemic.

### Sample Size Effects

The alpha coefficient for the EPSCALE in this study was high at 0.96, with inter-item covariance of 0.20, and with no case variability in reliability or covariance identified. The initial evaluation of the EPSCALE by Silverman et al. [25], reported a range of alpha coefficients from 0.80 to 0.89, depending on case. This study reports high reliability but did not identify a great deal of case to case variation. The Silverman study had 124 students, who completed 4 scenarios each, so a total of 496 scenarios were scored [25]. This study had a lower sample size of 46, with a total of 162 scenarios scored, from six individual scenarios. Therefore, the sample size for drawing conclusions relating to the reliability of the tool for individual scenarios was small. The majority of students in this study had 3 to 4 interactions, due to prevalent logistics, optimally more are required [50].

The reliability of OSCEs are widely debated [51–53]. Although the communication skills were specifically scored in this study and not combined with clinical skills, the small number of stations, paired with the small sample size may have limited the reliability of the results. OSCEs are known to need larger number of stations in order to produce results with high reliability [52], so, the findings of the quantitative component of this study have been interpreted with care and considered in light of the somewhat more positive qualitative data [54]. The decrease in communication scores was not significant for the erectile dysfunction and alcohol in pregnancy scenarios, which could imply a hint of case-to-case variation. We chose to study this men’s and women’s health module as we felt it would challenge students. This would be in line with the literature, which suggests that multiple factors impact on case-to-case variation in sexual health consultations, including gender, age and nationality within general medical practice [55–57]. All these variables were present within the scenarios in this intervention with male-female, female-male, older adults, younger adults and students from different nationalities included; however a larger sampling frame is required. The literature also signposts time limitations, which may inhibit sexual health communication, further highlighting the importance of considering time limits for consultations of this nature [57]. It is known that a large number of students completing a large number of cases is required to identify case-effects [58]. Thus, whilst the findings of this study were unexpected in terms of scoring, sample size effects were considered when refining initial programme theories and making educational recommendations.

### Educational Recommendations

This realist evaluation provides insight into the various contextual factors that may have an impact on the effect of SP training interventions. The focus group outcome findings illustrate that SP training can be a valuable approach. A time limit of five minutes per consultation is insufficient, and more time should be allowed for undergraduate students in training. Although some patient interactions could be completed within five minutes in practice, careful consideration of the complexity and the nature of the scenario is needed when determining an appropriate time limit, which may be on a case-by-case basis. Future SP interventions may be more successful if designed underpinned by a programme theory, with further consideration given to student contexts and intended outcomes. Theory-based design would also allow for more in-depth realist studies of such interventions. As dynamics seem to change adversely for students with an examiner present, consideration should be given as to whether communication based OSCEs can be scored remotely or need an examiner present, and if an examiner must be present, consideration must be given to the potential implications of this on communication scores. Lastly, students need to be clear of the formative intention of the ‘mock OSCE’, which may be facilitated by changing the nomenclature to ‘formative’ to emphasise the intention of assessment for learning.

### Strengths and limitations of this study

Strengths of this realist evaluation are that the initial programme theory was developed from a prior realist synthesis [18] and was conducted in line with RAMESES standards [22]. This evaluation was limited to a single iteration of the intervention for a single year group at a single institution, resulting in a relatively small sample size. The training intervention and the mock OSCE were non-equivalent in their conditions and set-up, with a faculty assessor, rather than a peer present at the mock OSCE, and this is somewhat of a limitation as it affected dynamics and, therefore, findings. In order to investigate communication further, conditions would need to be more controlled and consistent in both interventions, however, as this was a study of a natural intervention, this was not possible.

## Conclusions

This realist evaluation identified the contextual factors, which impact on how and the mechanisms by which SP communication training work for intermediate pharmacy students. SP training is a valuable communication skills training approach and emphasis should be placed on feedback and promotion of reflection. Time limits need to be considered in this context and adjusted to meet student needs, especially for students with lower levels of communication comfort and those communicating in languages different to their first language.

## Supplementary Information

**Additional file 1.**

## Data Availability

The datasets used and/or analysed during the current study are available from the corresponding author on reasonable request.

## Abbreviations

EPSCALE: Explanation and Planning Scale
OSCE: Objective Structure Clinical Examination
SP: Simulated Patient
