# Supplementary material for: A realist evaluation exploring simulated patient role-play in pharmacist undergraduate communication training
Source: BMC Med Educ. 2021 Jun 7;21:325. doi: 10.1186/s12909-021-02776-8 (PMC8180382; doi:10.1186/s12909-021-02776-8)
Supplement: Supplementary file 1 — Additional file 1. [file 12909_2021_2776_MOESM1_ESM.docx]

**Supplemental Information One: Focus Group Topic Guide**

**Opening**

1. Before the simulated patient (SP) session, what were your expectations of this session?
2. Did the SP session meet your expectations?
3. How did you feel about communication skills training before this session?
4. How do you feel about communication skills training following completion of this session?
5. What men and women’s health issue did you not know about before that were encountered in this session?

**What?**

1. Do you think that simulated patients are a useful method of training pharmacy students in communication skills?
2. If yes, why? If no, why not?

**Who?**

1. How do you feel about communicating with patients?
2. Have any of you worked in pharmacies before other than placement?
3. Have any of you any other part time jobs?
4. How did you feel about being videoed?
5. How did you feel about being observed by your peers?
6. How did you feel about the set-up of the debrief?

**How?**

1. How do you think SP session helped you learn?
2. If yes to Q14, what about the session do you think helped you learn?
3. If yes to Q14, how do you think the SP session promoted your own personal communication learning?

**In what circumstances?**

1. Do you think the setting of the SP session had any impact on its effect?
2. Do you think there are any barriers to the SP session effect?
3. Is there anything that you feel could have improved your learning?

**Closing**

1. Is there anything else that we have not yet talked about that you think would be useful or good to discuss?
